# Three Weeks of Pulmonary Rehabilitation Do Not Influence Oscillometry Parameters in Postoperative Lung Cancer Patients

**DOI:** 10.3390/medicina58111551

**Published:** 2022-10-28

**Authors:** Sabina Kostorz-Nosal, Dariusz Jastrzębski, Aleksandra Żebrowska, Agnieszka Bartoszewicz, Dariusz Ziora

**Affiliations:** 1 Department of Lung Diseases and Tuberculosis, Faculty of Medical Sciences in Zabrze, Medical University of Silesia in Katowice, 41-803 Zabrze, Poland; 2 Department of Physiological and Medical Sciences, Institute of Sport Sciences, Academy of Physical Education, 40-065 Katowice, Poland; 3 Independent Public Clinical Hospital No. 1, Medical University of Silesia, 41-800 Zabrze, Poland

**Keywords:** pulmonary rehabilitation, lung cancer, lung surgery, lobectomy, forced oscillation technique, oscillometry

## Abstract

**Simple Summary:**

Forced oscillation technique allows assessment of the respiratory system impedance, free from patient-dependent factors. Our study is the first to evaluate whether the implementation of physical activity has an influence on oscillometric results in patients after thoracic surgery due to lung cancer. The results indicate that despite the 6-min walking test and hand strength improvements, pulmonary rehabilitation has no impact on oscillometric values in this group of patients.

**Abstract:**

*Background*: Thoracic surgery is a recommended treatment option for non-small cell lung cancer patients. An important part of a patient’s therapy, which helps to prevent postoperative complications and improve quality of life, is pulmonary rehabilitation (PR). The aim of this study was to assess whether the implementation of physical activity has an influence on forced oscillation technique (FOT) values in patients after thoracic surgery due to lung cancer. *Methods*: In this observational study, we enrolled 54 patients after thoracic surgery due to lung cancer, 49 patients with idiopathic interstitial fibrosis (IPF), and 54 patients with chronic obstructive pulmonary disease/asthma–COPD overlap (COPD/ACO). All patients were subjected to three weeks of in-hospital PR and assessed at the baseline as well as after completing PR by FOT, spirometry, grip strength measurement, and the 6-min walk test (6MWT). *Results*: We observed differences between FOT values under the influence of physical activity in studied groups, mostly between patients after thoracic surgery and COPD/ACO patients; however, no significant improvement after completing PR among FOT parameters was noticed in any group of patients. Improvements in the 6MWT distance, left hand strength, and right hand strength after PR were noticed (*p* < 0.001, 0.002, and 0.012, respectively). *Conclusions***:** Three weeks of pulmonary rehabilitation had no impact on FOT values in patients after thoracic surgery due to lung cancer. Instead, we observed improvements in the 6MWT distance and the strength of both hands. Similarly, no FOT changes were observed in IPF and COPD/ACO patients after completing PR.

## 1. Introduction

Lung cancer has long been the most common malignant neoplasm in the world, with the highest mortality for both genders, accounting for approximately 18.4% of overall cancer mortality [1,2]. Nonetheless, at the time of diagnosis, only about 15% of cancers are operable [3]. Surgery may be the definitive available therapeutic strategy depending on the type of cancer, the stage of cancer, and other patient conditions (such as comorbidities and performance status). Complete tumor resection, if possible, is a recommended treatment option in non-small cell lung cancer patients [4,5]. However, thoracic surgery is fraught with the risk of postoperative pulmonary complications (PPCs), the most common of which are respiratory disorders (mainly respiratory failure) and cardiovascular events [5].

Among the methods of PPC prevention, chest physiotherapy plays a key role [5] because it increases mobility, breathing, and sputum clearance, as well as preserving lung function, especially when applied preoperatively [6,7,8]. Furthermore, the results of the latest studies clearly indicate the beneficial effects of pulmonary rehabilitation (PR) on the recuperation of functional efficiency, as well as improvements in the quality of life and life expectancy in this group of patients [9]. After thoracic surgery due to lung cancer, patients manifest obstructive disorders, so the rehabilitation model used in this group of patients corresponds to the rehabilitation program applied to patients with chronic obstructive pulmonary disease [10,11]. Some PPCs, such as atelectasis and fibrosis, may contribute to the prevalence of restrictive disorders that determine different lung function disorders. In these cases, it is possible that a completely different bedside manner, i.e., one with a rehabilitation scheme that brings greater benefits and advantages, should be applied.

Apart from quality-of-life questionnaires, the effectiveness of PR can also be objectively evaluated via lung function and exercise tests. Currently, spirometry is commonly performed for all patients [8]. However, this test requires forced expiration maneuvers that are highly dependent on the patient’s cooperation and effort. As a consequence, spirometry performance may prove to be problematic in children younger than 10 years and elderly populations older than 65 years [12,13]. Likewise, after thoracic surgery due to lung cancer, patients may be unable to correctly perform spirometry due to postoperative pain, lung stiffness, and fear associated with forced expiratory maneuvers, as well as the advanced age of diagnosis. These circumstances may have an effect on the obtained results.

In the case of the first two groups of patients (children and the elderly), the forced oscillation technique (FOT) has been successfully applied [14,15]. Thanks to the external excitation pressure generated by FOT devices and applied to airways during tidal breathing, oscillometry could be free from patient-dependent factors, such as coughing or pain, associated with recent thoracic surgery.

FOT allows one to assess respiratory, airway, lung parenchyma, or thoracic wall impedance. Therefore, an FOT device determines the resistance (R) and reactance (X) based on the response of the respiratory system to external stimuli [15,16]. R refers to the viscous resistance and is mostly dependent on airway diameter. The use of different frequencies allows one to separate airway resistance into total (R5, at 5 Hz), central (R19, at 19 Hz), and peripheral (R5–R19) resistance. The second impedance component is X, which is determined by elastic properties that dominate at low frequencies (X5) and inertia properties of the lung tissue that dominate at high frequencies (X19). The point when the value of X is equal to 0, at which elastic and inertia properties are equal, is called the resonant frequency (Fres).

Previous studies have indicated increased R5 and R5–20 and reduced X5 in obstructive lung diseases. Reduced X5 has mostly been observed in restrictive disorders [17,18,19,20,21]. The Fres parameter was found to be increased in both disorders. In contrast, only two studies so far have analyzed the impact of lobectomies on FOT values [22,23], as similar FOT abnormalities (however less expressed) were observed in chronic obstructive lung disease (COPD) patients. Additionally, there have been no reports assessing the impact of pulmonary rehabilitation on FOT parameters in postoperative lung cancer patients.

Therefore, the aim of this study was to assess whether the implementation of three weeks of in-hospital physical activity had any influence on FOT values in patients after thoracic surgery due to lung cancer. The secondary aim was to compare spirometry and effort test results before and after PR. These findings were further compared with a group of idiopathic interstitial fibrosis (IPF) and COPD or asthma–COPD overlap (ACO) patients undergoing the same 3-week PR program. The following article is presented in accordance with the STROBE reporting checklist presented in Table S1 (available as online Supplementary Materials).

## 2. Materials and Methods

### 2.1. Materials

**Figure 1:**
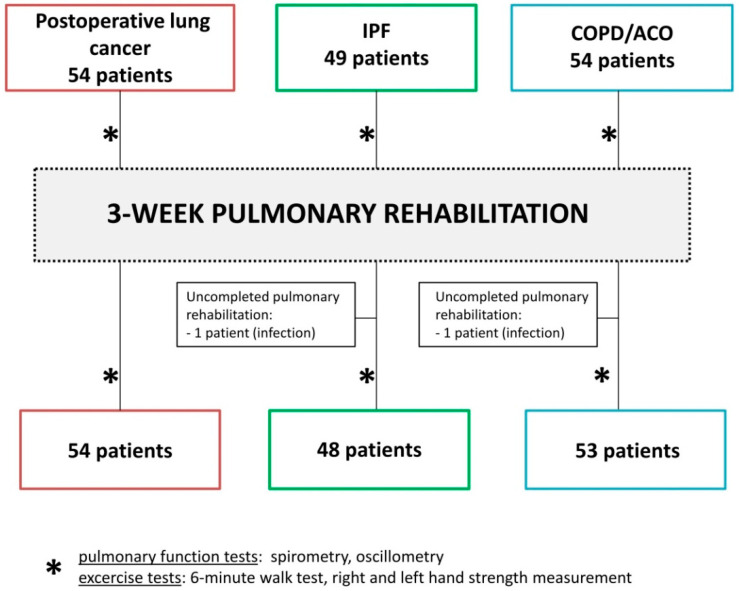
Study recruitment protocol.

Fifty-four patients after thoracic surgery due to lung cancer, forty-nine IPF patients, and fifty-four patients with preserved obstructive lung disease (COPD or ACO) were subjected to a 3-week inpatient rehabilitation program in the Department of Lung Diseases and Tuberculosis, Medical University of Silesia in Katowice between September 2018 and April 2022 (Figure 1). This was an observational study, so patients that matched inclusion criteria were consecutively enrolled in the project. The study was approved by the Bioethics Committee of Medical University of Silesia in Katowice (Act No. KNW/0022/KB1/85/I/17 from 19 December 2017) and was conducted in accordance with the Declaration of Helsinki. All participants provided written informed consent. The research was registered in the ISRCTN Trials Registry (ISRCTN31987937).

Inclusion criteria were defined as follows: the diagnosis of a disease based on actual guidelines for IPF [24,25], obstructive lung diseases [26,27], or non-small cell lung cancer (histologically or cytologically confirmed) after operative treatment within 6 months at stage I or II; a stable period of illness without infection/exacerbation during the previous 4 weeks; a distance in the 6-min walk test of no less than 250 m; and the ability and willingness to perform physical activity.

The exclusion criteria were as follows: patient’s refusal to engage in the rehabilitation program, infection/exacerbation during the previous 4 weeks, MRC Dyspnea Score > 3; unstable coronary artery disease, low performance level (Eastern Cooperative Oncology Group scale ≥ 3) [28], anemia (hemoglobin < 10 g/dL), and poor tolerability of pulmonary rehabilitation.

### 2.2. Physiological Measurements

All patients enrolled in the study performed lung function and exercise tests at the baseline and after 3 weeks of PR. A lung test apparatus (MES; Cracow, Poland) was used to perform spirometry in accordance with the manufacturer’s guidelines [29] and to assess forced expiratory volume during the first second (FEV_1_), forced vital capacity (FVC), and FEV_1_/FVC. The results are expressed as a percentage of predicted values. The grip strengths of the left and right hands were measured with a Meden-Inmed Baseline hydraulic hand dynamometer (Charder Medical, Taichung City, Taiwan) in compliance with the manufacturer’s guidelines [30]. Patients were asked to squeeze the dynamometer 3 times with each hand with all of their strength. Only the best score for each hand was included in the study. FOT measurements were evaluated with a Resmon Pro Full device (Restech Respiratory Technology SRL, Milano, Italy; marketed by MGC Diagnostics Cooperation, Saint Paul, MN, USA). These were performed over several seconds of tidal breathing through a mouthpiece in a sitting position. External small oscillatory pressures, applied to the airways, generated the respiratory system response and were used to record impedance (as appropriate): resistance (R, inspiratory and expiratory) at frequencies of 5 Hz, 11 Hz, and 19 Hz; reactance (X, inspiratory and expiratory) at frequencies of 5 Hz, 11 Hz, and 19 Hz; and resonant frequency (Fres). The impedance, resistance, and reactance results are expressed in cmH_2_O/L/s as percentages of the predicted values, and the Fres results are expressed in Hertz in accordance with the work of Oostveen et al. [15].

### 2.3. Pulmonary Rehabilitation

In-hospital PR was performed 5 days per week for a total of 3 weeks, under the supervision of a physical therapist and rehabilitation specialist. The program was carried out under the ATS/ERS recommendations [10]. Training intensity was based on the limit of the heart rate and the distance obtained during the 6MWT (6MWD), as previously described [31], and it included a combination of endurance and strength training [32].

The PR program consisted of 150 min per day: (1) aerobic exercises and flexibility training with elements of yoga and suspension UEU (Universal Exercise Unit allowing for relief and self-assisted exercises for 20 min) therapy; (2) breathing exercises (three times per day for 10 min); (3) lumbar and cervical stabilization exercises and equilibrium exercises (once per day for 20 min); (4) general rehabilitation gymnastics including stretching exercises, strengthening of the muscles of the upper and lower limbs and the back, as well as exercise for posture correction (once per day for 30 min); (5) continuous or interval endurance training (once per day for 30 min); (6) exercises on a stabilometric platform (once per day for 20 min); and (7) physiotherapy treatments depending on individual needs, e.g., ultrasound therapy or cryotherapy for back pain. Subsequent exercises of the daily PR program were performed within individual recovery periods.

This program was complemented by autogenic training, music therapy, and sessions with a psychologist (for 60 min per day).

### 2.4. Statistical Analysis

Statistica 13.3 (TIBCO Software Inc., Palo Alto, CA, USA, License SUM JPZ010A903827ARACD-F) was used for the statistical analysis. Descriptive statistics are presented as the means with standard deviation. The normality of distribution was checked with the Shapiro–Wilk test. The ANOVA Kruskal-Wallis test and Chi2 test were used to evaluate the differences in demographic data. Differences in lung function and exercise test results between groups, from baseline to the completion of pulmonary rehabilitation, and comparisons of parameter changes over time between groups were assessed with nonparametric analyses of longitudinal data, Analysis of Variance (ANOVA)-type statistics, and Nonparametric Analysis of Longitudinal Data (nparLD), using the nparLD library for R language in RStudio software [33]. Data are presented as medians with interquartile ranges. Significant results achieved in nparLD were evaluated in sequence via the pairwise multiple comparison Wilcoxon test. A *p*-value of 0.05 or less was considered statistically significant.

## 3. Results

All patients enrolled in the study (except for two due to infection) completed pulmonary rehabilitation and were re-evaluated with the same lung function and exercise tests used at the beginning (Figure 1). The PR program was carried out by the same staff, and there were no training modality changes during the study.

Demographic data are presented in Table 1. During the course of PR, no changes in the pharmacological treatment of underlying diseases occurred. None of the lung cancer patients enrolled in the study required analgesic treatment due to the recent thoracic surgery.

**Figure 2:**
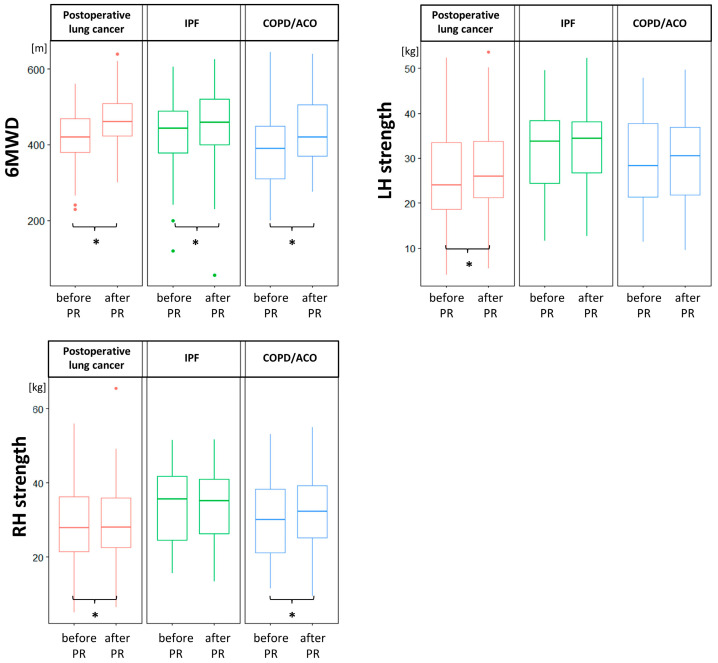
Significant improvements in the 6-min walk distance (6MWD), right hand (RH) strength, and left hand (LH) strength after completing 3 weeks of pulmonary rehabilitation (PR). The horizontal lines refer to medians, boxes to quartiles, and vertical lines to non-outlier range. IPF, idiopathic interstitial fibrosis; COPD, chronic obstructive pulmonary disease; ACO, asthma–COPD overlap; * *p* < 0.05.

All lung function and exercise test results, including FOT parameters, significantly differed between the studied groups of patients at the baseline and/or at the end of the PR program (Table 2 and Table 3). After three weeks of PR, we observed improvements in 6MWD in all group of patients, as well as hand strength improvement in COPD/ACO patients regarding the left hand, and in thoracic surgery patients regarding both hands (Figure 2). Other parameters did not change after completion of the rehabilitation program.

**Figure 3:**
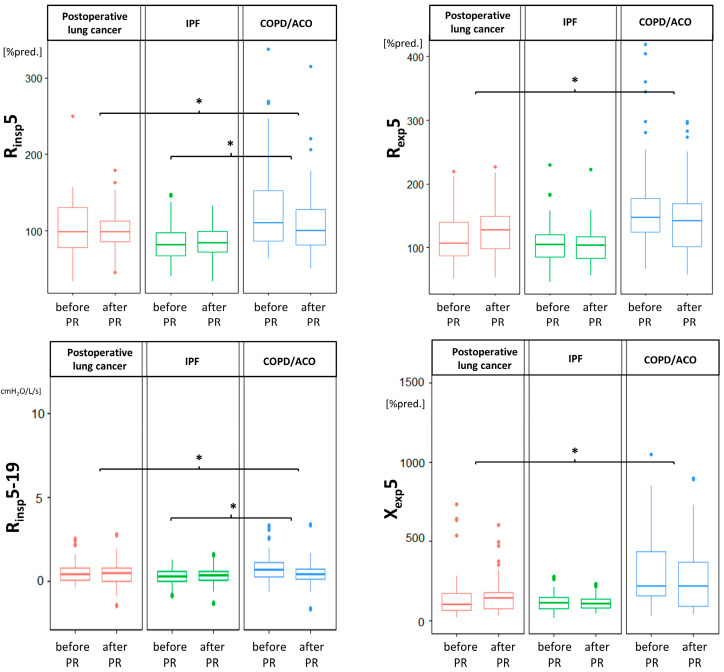
Differences between patient groups in terms of changes in resistance (R) and reactance (X) due to pulmonary rehabilitation (PR). The horizontal lines refer to medians, boxes to quartiles, and vertical lines to non-outlier range. IPF, idiopathic interstitial fibrosis; COPD, chronic obstructive pulmonary disease; ACO, asthma–COPD overlap; insp, inspiratory; exp, expiratory; * *p* < 0.05.

There were no differences in FOT values after PR (Table 3); however, we observed some significant differences between FOT changes in studied groups, mostly between patients after thoracic surgery and patients with obstructive lung disease (Figure 3).

## 4. Discussion

To the best of our knowledge, the impact of physical activity on oscillometry parameters in lung resection patients due to cancer has not yet been examined. According to our results, the FOT parameters in this group of patients remained stable after three weeks of pulmonary rehabilitation. As oscillometry is devoid of external factors that may have influenced the results (such as patient cooperation, muscle strength, and learning effect), we showed that three weeks of PR does not improve lung function, regardless of lung disease. Even if we observed significant disparities between changes in the FOT values due to PR in different groups of patients enrolled in the study (Table 3 and Figure 3), none of the studied groups demonstrated significant improvements or deteriorations with regard to oscillometric parameters.

However, these findings in no way imply that patients with lung diseases do not benefit from PR. We observed significant 6MWD improvements in all patients, as well as increases in right hand strength after training in COPD/ACO patients, and in both hands in patients after thoracic surgery due to lung cancer (*p* < 0.001, 0.016, 0.002, and 0.012, respectively; Table 2 and Figure 2).

These results may be considered to be rather disappointing when considering the well-documented positive impact of PR on quality of life, symptoms, muscle strength, and 6MWD [7,9,10,34,35]. However, these increases do not need to be consistent with lung function improvement, as studies have demonstrated PR-related improvements in skeletal muscle function and quality of life despite the absence of lung function improvements [36,37,38,39,40,41]. As COPD/ACO are systemic diseases, muscle dysfunction has a negative impact on these patients’ activities [42]. Lung cancer may also be considered to be a systemic disease with surgery-dependent consequences. Through peripheral muscle strength measurements, we showed that three weeks of PR may positively influence hand strength. These results are in concordance with previous meta-analyses showing improvements in muscle endurance and strength, as well as dyspnea reduction [43,44]. Although Brocki [45] reported improvements in respiratory muscle strength after two weeks of PR, regardless of the extent of the surgery or postoperative inspiratory muscle training, in this study, postoperative inspiration muscle training was connected to improvements in oxygenation.

Even though the authors of some studies have reported improvements in work breathing and lung volume influenced by PR in COPD patients, these have been argued to be caused by adjunctive therapies (such as medications, oxygen therapy, and exercise training) rather than breathing retraining alone [46]. As one PPC prevention method, perioperative PR has been recommended by the ERS [47,48]. A systematic review by Rodriguez-Larrad et al. [49] emphasized that pre-operative PR (compared to post-operative PR alone) is essential in functional capacity improvements and morbidity reductions. Contradictory data on pre-operative PR’s impact on lung function have been published. The authors of some studies observed no significant changes in spirometric results [40,41], while others reported improvements in FVC and FEV1 [50,51].

According to the ATS/ERS Guidelines, PR is one of the most effective non-pharmacological treatment options for COPD patients [34,52,53]. Dynamic lung hyperinflation, gas-exchange abnormalities, morphological alterations, and insufficient energy supplies are listed as pulmonary factors that may influence exercise limitations in COPD patients [54,55]. Until now, only two studies have presented analyses of the impact of PR on FOT measurements [31,56]. Zimmermann et al. [56] noticed that two 1 h sessions per week for a total of 16 sessions led to temporary improvements in X_insp_5 in COPD patients which were not observed at a 3-month follow-up; no other FOT changes were observed in that study. These improvements in lung volume were suggested to have resulted from reductions in ventilation heterogeneity. The differences between these results could derive from different PR settings, durations, and intensities. It is possible that the extension of PR duration would reveal improvements in FOT measurements. However, Zimmermann’s study was only conducted on 15 patients with no control group, so (as highlighted by the authors) the obtained results may not be representative and should be analyzed with caution [56].

In contrast, we previously reported that significant improvements in FVC were observed in patients with idiopathic interstitial pneumonia (IIP) subjected to three weeks of in-hospital PR, although, similarly, no changes in oscillometric results were noticed [31]. We reasoned that these results derived from better patient cooperation and compliance spirometry performance, improved diaphragm flexibility and strength, and the higher sensitivity of oscillometry in detecting SAD compared to spirometry [15,57].

It is commonly known that lung cancer surgery may be accompanied by many postoperative complications, with a frequency of between 2 and 40% [58,59,60,61], including pneumonia, atelectasis, acute respiratory failure, bronchospasm, pneumothorax, and prolonged air leaks. However, at the time of the admission to the Pulmonary Rehabilitation Department, we did not observe any serious pulmonary complications among postoperative patients. The authors of some studies have also addressed the subject of lung function deterioration after surgery and increased tissue resistance due to edematous changes and lung rigidity, which cause reduced lung compliance and decreases in ventilation due to intrathoracic pressure changes [22]. These factors contribute to the increased resistance of the thoracic wall, which, under normal conditions, represents one quarter of total airway resistance [62]. Local inflammation related to recent thoracic surgery may also lead to small airway disease [23].

Considering these results, lung function deterioration after thoracic surgery may be observed. Research on oscillometric changes in patients after lobectomy have consistently indicated increases in R5, R20, R5–R20, Fres, area of reactance (AX), and postoperative decreases in X5 and X11 [22,23]. Previous researchers have suggested that R depends on lung volume, as it was found to be correlated with decreases in VC and FEV_1_, as already described in this group of patients [63,64,65]. Additionally, a study performed by Drakou [66] indicated significant declines in FVC and FEV_1_ at one month after the thoracic operation was performed due to lung cancer diagnosis. Postoperative pulmonary function deterioration is mostly dependent on the extension of the surgery, so an R increase is coherent with VC and FEV1 decreases caused by lobectomy, according to volume reduction [65,67]. Additionally, there may be an impact of a postoperative anatomical repositioning or excursion of the remaining non-operated lobe which is even more pronounced in lobectomy compared to segmentectomy, due to its compensatory growth [68]. These results are in line with those of other studies, in which FVC was reported to improve over time as the stress applied by surgery declines [69,70]. However, the causes of postoperative lung function changes remain vague, as FOT changes were also described in surgery involving not the lungs, but the transurethral resection of a bladder tumor, under general anesthesia [71]. The authors of that study suggested that the observed lung function deterioration may have been caused by intraoperative lung injury triggered by intravascular fluid shifts, mechanical ventilation, and inhalational anesthetic drugs. Moreover, changes following lobectomy have been shown to be similar, regardless of concomitant lung diseases (COPD, IP, and CPFE) or their absence [22]. Similar findings were also obtained by Subotic et al. [72], who observed that there were no differences in lung function after lobectomy between patients with and without concomitant COPD.

As mentioned above, different lung diseases present different patterns of changes in FOT measurements [17,18,19,20,21]. Considering these conditions alone, more limitations in FOT results have been observed in patients after lobectomy compared to IPF patients, though they were less affected than COPD patients [23]. Interestingly, X11 decreases are more commonly seen in lobectomy patients than abnormalities in X5, though almost all previous studies considered only the X5 value. In a previous study, we observed only X11 decreases in IIP patients over three weeks, with or without PR [31]. However, in the current study, no changes in this parameter were observed, even in patients with IPF. Therefore, future studies are required to establish changes in oscillometric values as time passes, as well as to assess whether they are affected by physical activity.

Our study had several limitations. First of all, a small number of patients were enrolled in the study, which did not allow us to divide patients in terms of the extension of their lung surgery. Secondly, we did not include a control group consisting of postoperative lung cancer patients not subjected to PR. Thirdly, although lung cancer is more frequent in men, women presented greater willingness to undergo the pulmonary rehabilitation, and, therefore, applied more often for rehabilitation. Consequently, these circumstances had influenced the gender distribution and had contributed to the higher female participation. Finally, there was no possibility to extend the period of rehabilitation due to the National Health Fund limitations in Poland, which allow for a 3-week maximum in-hospital stay.

In conclusion, three weeks of pulmonary rehabilitation demonstrated no influence on FOT values in patients after thoracic surgery due to lung cancer. Instead, we observed improvements in 6MWD and the strength of both hands in this group of patients. Similarly, no FOT changes were observed in IPF and COPD/ACO patients. We believe that our data could facilitate the determination of the impact of a 3-week physical activity program on patient performance status and dispel doubts related to its influence on lung function.

## Figures and Tables

**Table 1:** Demographic data.

	Postoperative Lung Cancer (54)	IPF (48)	COPD/ACO (53)	*p*
Age (years)	67.4 ± 8.8	66.3 ± 6.1	65.9 ± 9.9	0.69
Sex (M/F)	23/31	34/14	32/21	0.014
BMI (kg/m^2^)	28.6 ± 4.5	27.2 ± 4.2	28.1 ± 6.0	0.308
Characteristics of the patient group	Histopathological diagnosis - adenocarcinoma: 27 pts - squamous cell carcinoma: 19 pts - non-small cell lung cancer: 4 pts - carcinoid: 2 pts - large cell carcinoma: 1 pt - metastases from breast cancer: 1 pt Type of operation (approximate percentage of the lung tissue removal) - lobectomy: 44 pts (16–26%) - bilobectomy: 4 pts (25–30%) - pneumonectomy: 3 pts (47–53%) - segmentectomy: 2 pts (5–6%) - wedge resection: 1 pts (8%) Neo-adjuvant therapy: 0 pts Adjuvant therapy: 23 pts	Antifibrotic treatment (77%): Nintedanib: 9 pts Pirfenidone: 28 pts GAP Index: Stage I: 31 pts (65%) Stage II: 15 pts (31%) Stage III: 2 pts (4%)	COPD: 41 pts (77%) ACO: 12 pts (23%)	-
Comorbidities				0.908
Cardiovascular diseases	42 (78%)	38 (79%)	39 (74%)	
Diabetes mellitus	13 (24%)	8 (17%)	14 (26%)	
Musculoskeletal disorders	22 (41%)	19 (40%)	31 (58%)	
Gastrointestinal disorders	13 (24%)	13 (27%)	11 (21%)	
Urogenital disorders	20 (37%)	16 (33%)	16 (30%)	
Psychiatric disorders	3 (6%)	3 (6%)	2 (4%)	
Smoking history				0.119
Current	4 (7%)	-	6 (11%)	
Previous	36 (67%)	30 (63%)	35 (66%)	
Never	14 (26%)	18 (38%)	12 (23%)	

IPF, idiopathic interstitial fibrosis; COPD, chronic obstructive pulmonary disease; ACO, asthma–COPD overlap; M, male; F, female; BMI, body mass index; pt(s), patient(s); GAP, the gender-age-physiology index for IPF patients.

**Table 2:** Lung function and exercise test results at the baseline and after 3 weeks of pulmonary rehabilitation (PR).

Parameter	Postoperative Lung Cancer (54)		IPF (48)		COPD/ACO (53)		npar LD Test		
	Baseline	After PR	Baseline	After PR	Baseline	After PR	Group	Time	Group: Time
FEV_1_ (%pred.)	76.31 ± 21.5	79.03 ± 19.8	82.98 ± 16.9	84.06 ± 17.9	57.55 ± 23.5	57.5 ± 22.7	<0.001 ^1,3^	0.409	0.313
FVC (%pred.)	92.33 ± 21.0	94.2 ± 21.7	80.78 ± 18.1	81.69 ± 18.9	80.62 ± 21.7	80.59 ± 21.2	0.003 ^1,2^	0.218	0.392
FEV_1_/FVC (%)	67.84 ± 8.8	67.81 ± 9.9	81.73 ± 6.9	81.92 ± 6.5	55.76 ± 14.4	56.12 ± 13.8	<0.001 ^4^	0.643	0.781
SpO_2_ (%)	95.88 ± 1.3	96.38 ± 1.7	94.77 ± 1.9	95.0 ± 2.4	94.68 ± 1.8	94.79 ± 1.5	<0.001 ^1,2^	0.123	0.592
6MWD (m)	415.7 ± 77.1	469.3 ± 71.5	426.6 ± 106.3	456.6 ± 105.5	382.3 ± 105.5	431.8 ± 91.7	0.032 ^1,3^	<0.001 ***	0.072
SpO_2_ after 6MWT (%)	92.53 ± 3.1	92.26 ± 3.4	84.83 ± 8.9	86.82 ± 7.5	89.76 ± 6.2	90.39 ± 4.8	<0.001 ^4^	0.491	0.263
RH strength (kg)	25.76 ± 10.6	27.19 ± 10.3	32.27 ± 9.8	32.54 ± 8.9	28.63 ± 10.4	29.57 ± 10.0	0.039 ^2^	0.002 *^,^**	0.007 ****
LH strength (kg)	28.37 ± 10.7	29.52 ± 11.2	34.19 ± 9.9	33.85 ± 9.3	30.33 ± 11.4	32.34 ± 10.5	0.007 ^2^	0.012 *	0.74

Results are expressed as means ± standard deviation. ^1^ Significant difference in lung cancer after thoracic surgery vs. COPD/ACO; ^2^ significant difference in lung cancer after thoracic surgery vs. IPF; ^3^ significant difference in IPF vs. COPD/ACO; ^4^ significant difference between all 3 groups; * significant improvement in lung cancer after thoracic surgery; ** significant improvement in COPD/ACO; *** significant improvement in all 3 groups; **** no significant difference based on the post hoc test. IPF, idiopathic interstitial fibrosis; COPD, chronic obstructive pulmonary disease; ACO, asthma–COPD overlap; FEV_1_, forced expiratory volume during the first second; FVC, forced vital capacity; SpO_2_, oxygen saturation; 6MWD, 6-min walk distance; 6MWT, 6-min walk test; RH, right hand; LH, left hand; group, differences between groups at the baseline and/or at the end of PR; time, changes in 3 weeks; group:time, and differences in changes after PR between studied groups.

**Table 3:** Forced oscillation results at the baseline and after 3 weeks of pulmonary rehabilitation (PR). IPF, idiopathic interstitial fibrosis; COPD, chronic obstructive pulmonary disease; ACO, asthma–COPD overlap; R, resistance; X, reactance; Fres, resonant frequency; _insp_, inspiratory; _exp_, expiratory; _tot_, total; group, differences between groups at the baseline and/or at the end of PR; time, changes in 3 weeks; group:time, differences of changes after PR between studied groups.

Parameter	Postoperative Lung Cancer (54)		IPF (48)		COPD/ACO (53)		npar LD Test		
	Baseline	After PR	Baseline	After PR	Baseline	After PR	Group	Time	Group:Time
R_insp_5 (%pred.)	104.97 ± 37.9	101.3 ± 28.7	85.79 ± 26.5	85.66 ± 22.6	129.08 ± 60.8	109.36 ± 46.9	<0.001 ^2,3^	0.09	0.029 ^1,3^
R_exp_5 (%pred.)	119.69 ± 44.5	127.99 ± 39.9	105.08 ± 34.8	103.38 ± 31.4	167.95 ± 80.1	148.89 ± 59.6	<0.001 ^4^	0.85	0.005 ^1^
R_insp_11 (%pred.)	108.65 ± 34.1	108.49 ± 24.8	92.91 ± 22.3	91.04 ± 17.6	129.07 ± 54.9	116.01 ± 43.0	<0.001 ^2,3^	0.408	0.11
R_exp_11 (%pred.)	122.34 ± 36.3	130.93 ± 34.7	113.76 ± 31.6	115.36 ± 28.2	149.97 ± 61.5	139.43 ± 47.3	<0.001 ^4^	0.224	0.068
R_insp_19 (%pred.)	95.99 ± 28.8	95.88 ± 21.0	81.94 ± 18.0	79.92 ± 14.6	109.48 ± 44.8	99.83 ± 31.5	<0.001 ^2,3^	0.393	0.206
R_exp_19 (%pred.)	100.98 ± 28.5	108.68 ± 28.9	95.5 ± 24.5	96.43 ± 22.0	118.46 ± 47.0	111.52 ± 33.4	0.005 ^2,3^	0.156	0.133
R_insp_5-19 (cmH_2_O/L/s)	0.51 ± 0.6	0.36 ± 0.7	0.25 ± 0.5	0.28 ± 0.6	0.77 ± 0.8	0.82 ± 3.2	0.021 ^1^^,3^	0.145	0.017 ^1,3^
R_exp_5-19 (cmH_2_O/L/s)	0.89 ± 1.0	0.8 ± 0.9	0.47 ± 0.6	0.3 ± 0.5	1.69 ± 1.1	1.2 ± 1.0	<0.001 ^4^	0.092	0.074
X_insp_5 (%pred.)	82.89 ± 41.8	87.37 ± 40.4	103.29 ± 54.2	110.33 ± 45.9	108.85 ± 58.5	106.89 ± 87.1	0.039 ^1,2^	0.414	0.089
X_exp_5 (%pred.)	147.54 ± 154.4	161.86 ± 122.8	116.4 ± 55.6	113.29 ± 49.0	318.26 ± 318.8	293.25 ± 294.8	<0.001 ^1,3^	0.656	0.048 ^1^
X_insp_11 (cmH_2_O/L/s)	−0.37 ± 0.5	−0.45 ± 0.6	−0.37 ± 0.4	−0.41 ± 0.5	−0.68 ± 0.7	−0.59 ± 0.6	0.029 ^1,3^	0.415	0.205
X_exp_11 (cmH_2_O/L/s)	−1.19 ± 1.5	−1.4 ± 1.3	−0.65 ± 0.5	−0.76 ± 0.7	−2.28 ± 1.5	−2.13 ± 1.5	<0.001 ^4^	0.117	0.025 *
X_insp_19 (cmH_2_O/L/s)	0.3 ± 0.5	0.27 ± 0.6	−0.26 ± 4.5	0.33 ± 0.5	0.02 ± 0.7	0.04 ± 0.6	0.002 ^1,3^	0.617	0.999
X_exp_19 (cmH_2_O/L/s)	−0.38 ± 1.0	−0.55 ± 0.9	0.02 ± 0.5	−0.03 ± 0.5	−1.07 ± 0.9	−1.01 ± 0.9	<0.001 ^4^	0.151	0.057
Fres (Hz)	15.84 ± 5.0	16.31 ± 5.6	15.2 ± 3.2	15.24 ± 3.6	19.08 ± 6.9	18.78 ± 6.3	0.003 ^1,3^	0.53	0.838

Results are expressed as means ± standard deviation. ^1^ Significant difference in lung cancer after thoracic surgery vs. COPD/ACO; ^2^ significant difference in lung cancer after thoracic surgery vs. IPF; ^3^ significant difference in IPF vs. COPD/ACO; ^4^ significant difference between all 3 groups; * no significant difference based on the post hoc test. IPF, idiopathic interstitial fibrosis; COPD, chronic obstructive pulmonary disease; ACO, asthma–COPD overlap; R, resistance; X, reactance; Fres, resonant frequency; _insp_, inspiratory; _exp_, expiratory; _tot_, total; group, differences between groups at the baseline and/or at the end of PR; time, changes in 3 weeks; group:time, differences of changes after PR between studied groups.

## Data Availability

Datasets analyzed during the current study are available in the Figshare (https://doi.org/10.6084/m9.figshare.20373930.v1 accessed on 26 July 2022).

## Supplementary Materials

The following supporting information can be downloaded at: https://www.mdpi.com/article/10.3390/medicina58111551/s1, Table S1: STROBE Statement—Checklist of items that should be included in reports of cohort studies.
